# Non-Amyloid Approaches to Disease Modification for Alzheimer’s Disease: An EU/US CTAD Task Force Report

**DOI:** 10.14283/jpad.2020.18

**Published:** 2020-04-06

**Authors:** Serge Gauthier, P. S. Aisen, J. Cummings, M. J. Detke, F. M. Longo, R. Raman, M. Sabbagh, L. Schneider, R. Tanzi, P. Tariot, M. Weiner, J. Touchon, B. Vellas, EU/US CTAD Task Force

**Affiliations:** 1 McGill Center for Studies in Aging, Verdun, QC Canada; 2 Alzheimer’s Therapeutic Research Institute (ATRI), Keck School of Medicine, University of Southern California, San Diego, CA USA; 3 Department of Brain Health, School of Integrated Health Sciences, University of Nevada Las Vegas (UNLV), Las Vegas, USA; 4 Cleveland Clinic Lou Ruvo Center for Brain Health, Las Vegas, NV USA; 5 Cortexyme, South San Francisco, CA USA; 6 Stanford University School of Medicine, Stanford, CA USA; 7 University of Southern California Keck School of Medicine, Los Angeles, CA USA; 8 Harvard University, Boston, MA USA; 9 Banner Alzheimer’s Institute, Phoenix AZ, USA; 10 University of California, San Francisco, CA USA; 11 Montpellier University, INSERM 1061, Montpellier, France; 12 Gerontopole, INSERM U1027, Alzheimer’s Disease Research and Clinical Center, Toulouse University Hospital, Toulouse, France

**Keywords:** Alzheimer’s disease, dementia, tau, tauopathy, neurotrophins, neuroinflammation, lifestyle intervention, photobiomodulation, neurostimulation, geroscience

## Abstract

While amyloid-targeting therapies continue to predominate in the Alzheimer’s disease (AD) drug development pipeline, there is increasing recognition that to effectively treat the disease it may be necessary to target other mechanisms and pathways as well. In December 2019, The EU/US CTAD Task Force discussed these alternative approaches to disease modification in AD, focusing on tau-targeting therapies, neurotrophin receptor modulation, anti-microbial strategies, and the innate immune response; as well as vascular approaches, aging, and non-pharmacological approaches such as lifestyle intervention strategies, photobiomodulation and neurostimulation. The Task Force proposed a general strategy to accelerate the development of alternative treatment approaches, which would include increased partnerships and collaborations, improved trial designs, and further exploration of combination therapy strategies.

## Introduction

**F**ollowing a discussion on lessons learned from clinical trials of amyloid-based therapies for Alzheimer’s disease (AD) (1), on December 4, 2019, the EU/US CTAD Task Force turned their attention to alternative approaches for disease modification. These strategies do not negate the validity of the amyloid hypothesis; indeed, recently discovered genetic evidence continues to support the centrality of amyloid in the neurodegenerative processes that lead to AD (2–4). However, genetic and other studies point to additional mechanisms and pathways both upstream and downstream of amyloidogenesis, which may provide druggable therapeutic targets with potential for disease modification.

Neuropathological and imaging studies confirm the complexity and heterogeneity of AD (5) Mixed pathologies are evident in most individuals with a clinical diagnosis of AD (6), and in early clinical studies of amyloid-targeting drugs, a significant proportion of trial participants were shown to have no detectable amyloid. Nonetheless, among putative disease-modifying AD drugs in clinical trials, 40% target amyloid either with small molecules or immunotherapies. Another 18% target tau. Other mechanisms targeted for disease modification include neuroprotection, anti-inflammatory effects, growth factor promotion, and/or metabolic effects (7). Additional trials are underway assessing non-pharmacological approaches to treat AD, including lifestyle interventions and neurostimulation.

## Anti-tau therapies

The microtubule-associated protein tau (MAPT, commonly referred to as tau) is the main constituent of the neurofibrillary tangles that are one of the two primary pathological hallmarks of AD. Its normal function is to stabilize microtubules and thus regulate intracellular trafficking, but in AD and other tauopathies, the protein undergoes post-translational modifications that lead to the development of a variety of oligomeric species, tangles, and neuropil threads that may be deposited as aggregates in specific brain regions, disrupting normal cytoskeletal function and protein degradation pathways (8). In the human brain, six isoforms of tau are present, which are classified as either 3R or 4R tau based on the number of repeat domains. Approximately equal levels of 3R and 4R tau are expressed in the normal brain; however, 3R:4R tau imbalances are seen in brains of individuals with tauopathies. In AD, isoform imbalances vary across brain regions and disease progression.

Unlike levels of amyloid beta protein (Aβ), which correlate poorly with cognition, tau levels are associated with both neurodegeneration and cognitive deficits (9). Tau pathology has been shown to follow a characteristic progression pathway in the brain, starting in areas responsible for learning and memory before spreading to cortical areas involved in other cognitive functions (10).

The complex progression of tau pathological events provides multiple potential opportunities for intervention. Anti-tau drugs in development target tau expression, aggregation, degradation, protein modifications (e.g. phosphatase modifiers, kinase inhibitors), microtubule stabilization, and extracellular tau inter-neuronal spread (8). As of February 2019, clinical trials were underway for 17 tau-targeting drugs — seven small molecules and 10 biologics (7). Only one drug, LMTX (TRx0237) — a reduced form of methylene blue, and a tau protein aggregation inhibitor -- is currently being tested in a Phase 3 trial in early AD at 8 — 16 mg/day doses versus placebo (NCT03446001). This trial follows two Phase 3 trials in mild and mild to moderate AD (NCT01689246, NCT01689233) and a trial in behavioral variant FTD (NCT01626378) with higher doses, which showed negative results in the primary analysis of clinical efficacy. Biogen has a Phase 2 study underway of the anti-tau agent BIIB092 (gosuranemab) in participants with MCI due to AD or mild AD (NCT03352557). Phase 2 studies in biologically defined populations are also being conducted. For example, Roche/Genentech is conducting two Phase 2 studies of the anti-tau monoclonal antibody semorinemab in participants with prodromal or probable AD confirmed by amyloid positron emission tomography (PET) or cerebrospinal fluid (CSF) testing (NCT03828747). Clinical trials of anti-tau therapeutics have been conducted in other tauopathies, although two recent Phase 2 studies of anti-tau monoclonal antibody therapies (Abbvie’s AABV-8E12 and Biogen’s gosuranemab) in participants with progressive supranuclear palsy (PSP) were recently terminated for lack of efficacy (NCT2985879 and NCT03068468, respectively). Non-clinical studies of innovative anti-tau therapies are underway, such as a study that uses engineered tau-degrading intrabodies to target intracellular tau (11).

It is also theoretically possible that early anti-amyloid intervention may attenuate or even preclude downstream effects on tau. That is, non-tau-based treatments could have implications for tau and tangles.

Several challenges face developers of tau-based therapeutics. For tau reduction approaches, it is not known how much reduction is needed, how quickly and safely it can be accomplished, when different interventions might be effective during the course of the disease, and how long drug levels must be maintained to get an effect. Tau biology is complicated with numerous fragments and post-translational modifications associated with tauopathies, yet it remains unclear which tau species are toxic. Moreover, the targets, mechanisms and cellular locations through which such tau species promote degeneration remain to be identified. These issues make the design of clinical trials especially complicated and highlight the need for better tau biomarkers. Recent progress made in the development of tau ligands for PET may improve the efficiency of clinical trials, since tau-PET enables early diagnosis and tracking of disease progression, identifying individuals at risk for faster cognitive decline, and rapidly assessing pharmacodynamic effects of treatments (12). Plasma levels of total tau (t-tau) and neurofilament light (NfL) have been developed as biomarkers of neurodegeneration (13). Still needed are biomarkers that distinguish 3R from 4R tau and that quantify the many different tau species.

## Neurotrophic strategies

The neurodegeneration that occurs in AD results from a complicated molecular and biochemical signaling network, likely triggered by Aβ and eventually leading to synaptic dysfunction, loss of dendritic spines, and neurite degeneration (14). Growth factors called neurotrophins regulate neuronal survival, development, and function by binding to cell surface receptors. The signaling networks regulated by these receptors have extensive overlap with those associated with neurodegeneration and modulation of neurotrophin receptors has thus been proposed as a potential therapeutic strategy (15). The Longo lab and others have zeroed in on the p75 neurotrophin receptor (p75NTR) as a therapeutic target for AD. Their working hypothesis, supported by human genomic and proteomic data, along with animal studies is that the p75NTR modulates the complex AD degenerative signaling network and that downregulating its signaling renders oligomeric Aβ unable to promote degeneration (16, 17).

Longo and colleagues have developed small molecule ligands that bind to p75NTR, activate survival-promoting signaling, and prevent Aβ-induced neurodegeneration and synaptic impairment (18). One molecule in particular, LM11A-31, has been shown to block Aβ-induced tau phosphorylation, misfolding, oligomerization and mislocalization; reverse late-stage spine degeneration; reverse synaptic impairment; prevent microglial dysfunction; and in wildtype mice suppress age-related basal forebrain cholinergic neuron degeneration (18–20). There is evidence that dendritic spine preservation is associated with cognitive resilience (21).

A Phase 2a pilot study sponsored by PharmatrophiX Inc. and funded in part by the National institute on Aging (NIA) and the Alzheimer Drug Discovery Foundation is underway, testing oral LM11A-31 in participants with mild-to-moderate AD and amyloid positivity assessed by CSF Aβ screening (NCT03069014). With an expected completion in the third quarter of 2020, the trial will assess safety and tolerability as well as cognitive, clinical, biomarker, and imaging exploratory endpoints. LM11A-31 may be effective in other disorders such as Huntington’s disease (22), diabetes-induced macular oedema (23), and traumatic brain injury (24).

## Anti-microbial and anti-inflammatory strategies

Neuropathological studies of the AD brain show not only amyloid plaques and tau-based tangles but neuroinflammation as well. Indeed, according to the innate immune hypothesis, plaques, tangles, and neuroinflammation orchestrate an innate immune response that has evolved to protect the brain against microbial infection, with Aβ itself acting as an antimicrobial peptide (AMP) in the brain (25, 26). This hypothesis suggests that subclinical microbial infections in the brain rapidly ‘seed’ Aβ to trap microbes, and that this process drives Aβ neurotoxicity and opsonization (i.e, an ‘eat me’ signal for microglia to remove axons and synapses) (25). Tangles form in response to microbe invasion to block neurotropic microbe spread. AD risk genes are implicated in the innate immune protection hypothesis, which posits that AD-associated genetic risk variants were evolutionarily conserved to keep Aβ deposition, tangle formation, and gliosis/neuroinflammation on a ‘hair trigger’ as a means of protecting a subset of the human species in the advent of a major epidemic of brain infection.

The molecular pathways involved in these processes provide multiple potential therapeutic targets, including the use of anti-viral drugs, antibiotics, blockade of toxic microbial products, and immunization for prevention of subclinical infections; secretase inhibitors and immunotherapies to prevent Aβ seeding; kinase or phosphatase inhibitors to prevent the development of pathological forms of tau, and anti-inflammatories to suppress neuroinflammation. Gut microbiota may also play a role in AD pathogenesis by disrupting neuroinflammation and metabolic homeostasis, thus representing another potential intervention target (27).

One example of a bacterial hypothesis and associated strategy is based on the discovery of the bacterium Porphyromonas gingivalis (Pg), most commonly associated with periodontitis, in the brains of AD patients. Toxic virulence factors from the bacterium, proteases called gingipains, have been identified in AD brains, and gingipain levels correlated with tau and ubiquitin pathology. Oral infection of mice with Pg resulted in brain colonization, increased Aβ1-42, and loss of hippocampal neurons, effects that were blocked by COR388, a small-molecule irreversible lysine- gingipain inhibitor. COR388 significantly lowered markers of inflammation in plasma as well as AD-associated APOE fragments in CSF in a small Phase 1b study in mildmoderate AD patients (28), and a large Phase 2/3 study is underway with an interim readout expected in Q4 2020 and topline data in Q4 2021 (NCT03823404).

A retrospective cohort study showed that Herpes simplex virus (HSV)-infected subjects had a nearly 3-fold increased risk of AD but that treatment with anti-viral drugs such as acyclovir brought risk to noninfected levels (29). There is an ongoing phase 2 trial of valacyclovir for patients with mild AD and positive titers for HSV1 and HSV2 (NCT03282916). Trials in AD using doxycycline and minocycline did not show efficacy (30).

Anti-inflammatory strategies are also being pursued. A Phase 2 study underway in participants with late mild cognitive impairment (MCI) or early AD aims to protect neurons against oxidative stress using two small molecule drugs -- tauroursodexycholic acid (TUDCA) and sodium phenylbutyrate -- repurposed by Amylyx Pharmaceutical as AMX0035 (NCT03533257). Yet another Phase 3 study sponsored by AZTherapies, Inc. aims to reduce neuroinflammation by converting microglia from a proinflammatory to phagocytic state to promote clearance of Aβ by using a combination of two marketed drugs, cromolyn and ibuprofen, known as ALZT-OP1 (NCT02547818) (31).

## Lifestyle intervention strategies and other non-pharmacological approaches

Multiple epidemiological studies in Europe, the United States, and Canada investigating an observed decline in the prevalence of dementia in recent years have suggested that dementia may be preventable by targeting lifestyle risk factors such as diabetes, hypertension, obesity, physical inactivity, smoking, depression, low education, and social isolation (32). Clinical studies are now beginning to support this assertion. The Systolic Blood Pressure Intervention Trial -- Memory and Cognition in Decreased Hypertension (SPRINT MIND) study suggested that intensive blood pressure control may reduce the risk of probable dementia and mild cognitive impairment (MCI), although the results were not statistically significant, in part because the SPRINT trial was terminated early based on the significant benefits of blood pressure control on cardiovascular outcomes. The study may have been underpowered for cognitive endpoints (33). Further study is warranted given that a 10-year study in France showed that hypertension was associated with poorer cognition in middle-aged individuals (34).

Multi-domain strategies have focused on lifestyle factors. For example, the Finnish Geriatric Intervention Study to Prevent Cognitive Impairment and Disability (FINGER) trial demonstrated improved or stabilized cognitive function in participants that adhered to an intervention combining diet, physical exercise, cognitive training, and vascular risk monitoring (35). The Multidomain Alzheimer Prevention Trial (MAPT)d tested an intervention combining cognitive and physical intervention along with omega-3 polyunsaturated fatty acid supplementation in frail, non-demented, community dwelling adults (36, 37). While MAPT failed to demonstrate significant slowing of cognitive decline, subgroup analyses suggested that individuals with low plasma levels of docosahexaenoic acid (DHA, an omega-3 fatty acid) have more cognitive decline, which appeared to be normalized with omega-3 supplementation(38). The benefits of omega-3 supplementation appeared to be greater in amyloid-positive individuals and in those with increased cardiovascular risk scores (39, 40). Based on the results from FINGER, MAPT, and other multidomain intervention studies, many additional studies are planned, including worldwide FINGERS studies (WW-FINGERS), a network of studies throughout the world that are adapting the multidomain strategies of the FINGER trial to different populations (41).

In addition to physical and cognitive activity, other non-pharmacological strategies are being investigated for their potential to slow cognitive decline and prevent dementia. For example, photobiomodulation (PBM) has been shown to be neuroprotective. In animal models PBM improved memory and normalized markers of AD, oxidative stress and neuroinflammation (42). A pilot study is now underway in participants with probable AD (NCT03405662).

Non-invasive neurostimulation with techniques such as repetitive transcranial magnetic stimulation (rTMS) has been proposed as a treatment for AD (43). Other technological approaches including assistive technologies, smart technologies, and telemedicine may improve the treatment and care of people with AD.

## GeroSciences

Given that aging is the major risk factor for AD, therapeutic strategies aimed at the diseases of aging (e.g., frailty) may slow cognitive decline and the development of dementia (44) Considerable research is underway to investigate the relationship between biological aging and neurodegenerative disease. These efforts have coalesced in the emerging field of geroscience (44), which explores whether the physiological hallmarks of aging such as mitochondrial dysfunction, loss of proteostasis, increased cellular senescence, and stem cell exhaustion may contribute to the development of AD pathology and neurodegeneration (45). Identification of biomarkers of aging and elucidation of how the molecular pathways of aging and AD intersect could advance the identification of novel therapeutic targets and next-generation therapies, such as the use of mesenchymal stem cells (46). The links between aging and AD are being explored as one element of the INSPIRE Research Initiative (Barreto JFA in press).

## Conclusions/moving forward

While the AD drug development pipeline continues to be dominated by Aβ-targeting therapies, there is increasing recognition that addressing the complexity of AD may require multiple agents and may need to start in early disease stage before pathology becomes irreversible. A “deep biology” view, such as that proposed by advocates of p75NTR modulation, posits that key ‘hub’ targets may enable modulation of multiple mechanisms (e.g. resilience to both Aβ and tau) and that key components of pathology could be reversible (e.g. spines, synaptic function). A single treatment could thus promote synaptic function and slow progression and prevent upstream tau aggregation and oligomer formation.

Given the importance of tau in the development of AD, and reflecting the recently proposed Research Framework (47), CTAD Task Force members advocated assessment of both Aβ and tau levels in all clinical trials. The A-T+N+ AD phenotype is common and should be targeted for anti-tau trials. A suggestion was made to name this phenotype Dementia Associated and Neurofibrillary tangle Neuroimaging Abnormality (DANNA). Tau imaging may provide a biological outcome, at least in Phase 2 studies, although the Task Force recognized that amyloid and/or tau PET imaging adds substantial subject and trial burden and cost. Other suggestions that could accelerate the development of anti-tau therapies include using basket designs that include participants with other tauopathies such as frontotemporal degeneration (FTD), progressive supranuclear palsy (PSP), and corticobasal degeneration (CBD). While such trials would include participants with heterogeneous presentations, an outcome assessment such as Goal Attainment Scaling (GAS) could enable capture of clinically meaningful outcomes from diverse participants. This tool enables patients, caregivers, and clinicians, to set goals for treatment using a standardized guided interview, followed by an assessment of whether those goals have been attained (48, 49).

The Task Force suggested that combination therapy may be required to tackle such a complex disease as AD (50). They also advocated employing other innovative clinical trial methodologies to accelerate development of alternative approaches.

The Task Force proposed a general strategy to accelerate the development of alternative treatment approaches, which would include:

- Increased partnerships in the pre-competitive space with increased sharing of granular level data, shared biomarkers, statistical approaches, information on site performance
- Innovative trial design
- More collaborative approaches to recruitment and retention of participants for clinical trials with a focus on participation of representative populations.
